# Sarcomatoid Renal Cell Carcinoma: A Clinical Review

**DOI:** 10.3390/diagnostics16172711

**Published:** 2026-08-25

**Authors:** Piotr Remiszewski, Anna Szumera-Ciećkiewicz, Anna M. Czarnecka

**Affiliations:** 1 Department of Soft Tissue/Bone Sarcoma and Melanoma, Maria Sklodowska-Curie National Research Institute of Oncology, 02-781 Warsaw, Poland; 2 Medical Faculty, Medical University of Warsaw, 02-091 Warsaw, Poland; 3 Outpatient Chemotherapy Department, Maria Sklodowska-Curie National Research Institute of Oncology, 02-781 Warsaw, Poland; 4 Biobank, Maria Sklodowska-Curie National Research Institute of Oncology, 02-781 Warsaw, Poland; 5 Department of Experimental Pharmacology, Mossakowski Medical Research Institute, Polish Academy of Sciences, 02-106 Warsaw, Poland

**Keywords:** sarcomatoid renal cell carcinoma, immune checkpoint inhibitors, sarcomatoid dedifferentiation, nivolumab, pembrolizumab, adjuvant therapy, prognosis, imaging

## Abstract

This review summarises current evidence on renal cell carcinoma (RCC) with sarcomatoid dedifferentiation (sRCC), with an emphasis on diagnosis, prognostic stratification, and treatment. Sarcomatoid dedifferentiation is characterised by high-grade spindle cell morphology, occurs in 2–26% of all RCC patients, predominantly in the clear cell subtype (~70%), and is WHO/ISUP grade 4 regardless of extent of involvement. Thus, sRCC represents a pathological transformation rather than a distinct entity. Diagnosis requires biopsy or nephrectomy; immunohistochemical (IHC) retention of PAX8, cytokeratins, and vimentin alongside loss of subtype-specific markers distinguishes sRCC from primary renal sarcoma. On CT, sRCC typically presents as a large, heterogeneous mass with extensive necrosis; 18F-FDG PET/CT carries independent prognostic value, though no feature is pathognomonic. Sarcomatoid features are approximately five times more prevalent in patients with metastatic vs. localised disease (~20 vs. ~4%), and their identification should prompt risk stratification. Median overall survival (OS) in metastatic sRCC is 5.9–13.3 months, though recent data suggest improvement with novel therapies. Sarcomatoid dedifferentiation is characterised by enrichment of alterations in TP53, BAP1, CDKN2A/B, and NF2 on a background of founder RCC mutations, generating an immune-inflamed tumour microenvironment with elevated CD8+ T cell infiltration and PD-L1 expression that may underpin the heightened sensitivity of sRCC to immune checkpoint inhibitors (ICI). Surgery remains the cornerstone in localised disease, although recurrence rates approach 80% within two years. Adjuvant pembrolizumab significantly improved disease-free survival in high-risk localised RCC with sarcomatoid features (KEYNOTE-564; NCT03142334). In the metastatic setting, nivolumab plus ipilimumab and pembrolizumab plus axitinib are preferred first-line regimens.

## 1. Introduction

Renal cell carcinoma (RCC) with sarcomatoid dedifferentiation, also known as sarcomatoid RCC (sRCC), is not a distinct histological entity but rather a high-grade, dedifferentiated state that may arise from any RCC subtype [1,2]. Any RCC with sarcomatoid dedifferentiation is classified as WHO–International Society of Urological Pathology grade 4 [2]. Dedifferentiation is characterised by the presence of spindle-shaped cells resembling sarcoma and is associated with worse prognosis. While sRCC can occur in localised disease, diagnosis is usually delayed, with metastases already established [3,4,5].

Unlike sarcomas, which are of mesenchymal origin, sRCC originates from epithelial renal tumours undergoing sarcomatoid dedifferentiation, frequently via epithelial–to-mesenchymal transition (EMT) [6,7,8]. Therefore, once a confirmed diagnosis of sRCC is established, management should follow RCC—rather than sarcoma—treatment paradigms, as its molecular drivers and therapeutic targets are more similar to those of high-grade RCC than those of soft tissue sarcomas [6,7,8]. Due to the distinct molecular profile and aggressive behaviour of sRCC, treatment strategies must be tailored to the disease stage. This review addresses the management of localised and metastatic sRCC separately.

This narrative review was conducted by searching PubMed/MEDLINE, Embase, and the Cochrane Library (inception to May 2026) using the terms ’sarcomatoid renal cell carcinoma’, ’sarcomatoid dedifferentiation’, and ‘RCC sarcomatoid’, combined with ‘diagnosis’, ‘prognosis’, ‘immunotherapy’, ‘surgery’, and ‘molecular’. No date restriction was applied. Randomised controlled trials, prospective cohorts, and large retrospective series (*n* ≥ 30) were prioritised; post hoc and single-arm analyses were identified as such throughout the text. Conference abstracts are cited only where peer-reviewed data are unavailable. The PRISMA guidelines were not applied due to the narrative design.

## 2. Incidence and Presentation

Sarcomatoid features can occur in 2–26% of all RCC subtypes, most commonly in clear cell RCC and less frequently in papillary and chromophobe histologies. Dedifferentiation is much more common in metastatic disease, with approximately 20% occurrence vs. 4% in localised disease [9]. Seventy per cent of sarcomatoid changes arise on a clear cell background. Within clear cell tumours, the frequency of sarcomatoid transformation is 5–13%, whereas among non-clear-cell subtypes it is 2–7% in papillary tumours, 9–13% in chromophobe tumours, 11–26% in unclassified tumours, and 29% in collecting duct carcinomas [10,11,12]. Clinically, these tumours present at a median age of 58–70 years, predominantly in men (male-to-female ratio 1.3:1 to 2:1 [4,11,13,14,15]) and in around 90% of all symptomatic cases at diagnosis [11,16,17,18]. Between 45% and 64% of patients are diagnosed with stage IV disease, indicating metastatic disease, with the most common sites being the lungs (40–67%), bones (10–36%), and liver (12.6–23.0%) [5,9,19,20,21,22]. sRCC tumours are often described as large, with many studies reporting that they are significantly larger than other RCC subtypes [18,20,23]. Some studies report median sizes ranging from 8.3 cm to 10.5 cm. Approximately 36.4% of sRCC tumours exceed 10 cm in maximal diameter [20,23]. Larger size is considered a negative prognostic factor. A definitive diagnosis of sarcomatoid RCC necessitates histopathological examination, as there are no pathognomonic imaging findings [23,24]. Radiographically, sRCC is comparable to advanced RCC, though it frequently exhibits extensive necrosis. On MRI, it may manifest as a heterogeneous renal mass with regions of low T2 signal intensity, a feature that remains non-specific [24,25].

## 3. Diagnosis

The diagnostic process is complicated by the focal, uneven intra-tumoural distribution of the sarcomatoid component, which predisposes to sampling error on needle biopsy. Most frequently, the sarcomatoid differentiation is diagnosed after surgery [26]. The sarcomatoid component is characterised by spindle-shaped cells, often with increased pleomorphism and high mitotic activity [27]. Standard biopsy techniques have low sensitivity for sRCC, and studies show that multi-quadrant biopsies (obtaining multiple samples from different areas within the tumour) can improve detection rates, with 86.7% detection compared to 25% with the standard technique [28,29]. Consequently, a definitive diagnosis is contingent on histopathologic and immunohistochemical (IHC) evaluation post-nephrectomy, in which markers such as keratin, vimentin, and high Fuhrman grades (typically grade IV) are frequently observed [28,30].

The most important differential diagnoses include the following [14,18,26,28,30,31]:

- Clear cell RCC with early spindle cell change (reactive-appearing stroma without true malignancy; look for clear cell component) [32];
- Angiomyolipoma (epithelioid variant) (triphasic or epithelioid; HMB45+, Melan-A+, SMA+, PAX8-, cytokeratin-; less pleomorphic) [31];
- Sarcomatoid urothelial carcinoma (history of lower tract UC, renal pelvis location, CIS or papillary remnants; p63+, GATA3+, uroplakin II+, PAX8-, though PAX8 may be focally + in high-grade cases) [33,34];
- Mucinous tubular and spindle cell carcinoma (bland spindle cells, mucinous stroma, anastomosing tubules; lacks high-grade features of sRCC; rare sarcomatoid change reported) [35];
- Sarcomatoid adrenal cortical carcinoma (adrenal origin; SF1+, inhibin+, calretinin+, Melan-A+, synaptophysin+, PAX8-) [31];
- Primary renal sarcoma (no epithelial component on extensive sampling; most often leiomyosarcoma fascicles, cigar-shaped nuclei, desmin+, PAX8-) [31,35,36];
- Retroperitoneal sarcoma involving kidney (commonly liposarcoma; PAX8-; no epithelial component) [3,11,35,37].

In the current WHO framework, ‘sarcomatoid renal cell carcinoma’ is not regarded as a distinct histological entity [38]. Sarcomatoid morphology is defined as a high-grade dedifferentiated component that may arise in any subtype of renal cell carcinoma, including clear cell, papillary, chromophobe, collecting duct, medullary, MiT family translocation-associated RCC, and unclassified RCC. The diagnostic task is therefore not to classify a tumour as ‘sarcomatoid carcinoma of the kidney’ but to identify the underlying RCC subtype whenever possible and to document the presence and extent of sarcomatoid differentiation [26,38,39].

Histologically, sarcomatoid differentiation is characterised by an abrupt or gradual transition from conventional RCC to a spindle cell and/or pleomorphic malignant component resembling high-grade sarcoma. The tumour may show fascicular, storiform, herringbone-like, or undifferentiated growth; marked nuclear pleomorphism; frequent atypical mitoses; necrosis; and desmoplastic or myxoid stroma. Heterologous elements are uncommon but may occur. Recognition is primarily morphological; immunohistochemistry is supportive, particularly in limited biopsies, metastatic sites, and tumours in which the conventional epithelial component is absent or minimal [28,30,31].

From a grading perspective, sarcomatoid differentiation is automatically a WHO/ISUP grade 4 feature, irrespective of the percentage of the tumour involved. There is no accepted minimum quantitative threshold: even a small, unequivocal sarcomatoid component should be reported. Where feasible, the estimated percentage of sarcomatoid differentiation should be provided because it improves clinicopathological characterisation and may have prognostic relevance. The ISUP grading recommendations and structured reporting datasets support explicit reporting of sarcomatoid and rhabdoid morphology as adverse parameters [38,39,40,41,42].

Immunohistochemistry should be used as a problem-oriented panel rather than isolated markers. PAX8, cytokeratins, and EMA help establish renal epithelial differentiation; CAIX, CD10, CK7, AMACR, and CD117 help refine the RCC subtype; GATA3, p63, p40, and uroplakins separate urothelial carcinoma; SMA, desmin, and h-caldesmon evaluate smooth muscle differentiation; HMB45, Melan-A, and cathepsin K support PEComa; SOX10 and S100 support melanoma; and MDM2/CDK4 raise the possibility of dedifferentiated liposarcoma. The key diagnostic principle is that sarcomatoid differentiation should be considered a high-grade morphological transformation of RCC, rather than a stand-alone renal tumour category. The pathology report should clearly communicate the underlying RCC subtype if identifiable, the presence of sarcomatoid component, and its estimated extent when assessable [28,30,31] (Table 1).

### Imaging

Imaging plays a central—though not pathognomonic—role in the initial evaluation of sRCC. On contrast-enhanced CT, sRCC typically presents as a large renal mass (mean diameter ~77–100 mm) with marked tumour heterogeneity, extensive central necrosis, and prominent peritumoral neovascularity; these features collectively distinguish sRCC from conventional clear cell RCC with reasonable discriminatory accuracy (AUC 0.81 by texture analysis) [45]. Renal sinus invasion, perinephric fat extension, and renal vein or inferior vena cava involvement are frequently evident on staging CT, reflecting the tumour’s aggressive locoregional behaviour. On MRI, sRCC demonstrates an infiltrative morphology, heterogeneous T2 signal, and irregular enhancement with non-enhancing necrotic foci; low T2 signal in the solid component may reflect the high nuclear-to-cytoplasmic ratio of the sarcomatoid spindle cell population [23,24]. Importantly, no imaging feature is specific for sarcomatoid differentiation—histological confirmation remains mandatory [41,42]. Furthermore, 18F-FDG PET/CT is not recommended for routine diagnosis of primary renal masses; however, in sRCC specifically, FDG avidity is significantly higher than in conventional ccRCC (with SUVmax cutoff 5.4 the sensitivity, specificity, and accuracy were 100%, 82%, and 87%, respectively), and metabolic parameters—particularly total lesion glycolysis (TLG)—carry independent prognostic value for disease-free survival and overall survival [46]. PET/CT may therefore be considered selectively in patients with high-risk or metastatic disease for staging, prognostication, and assessment of treatment response [45].

**Figure 1:**
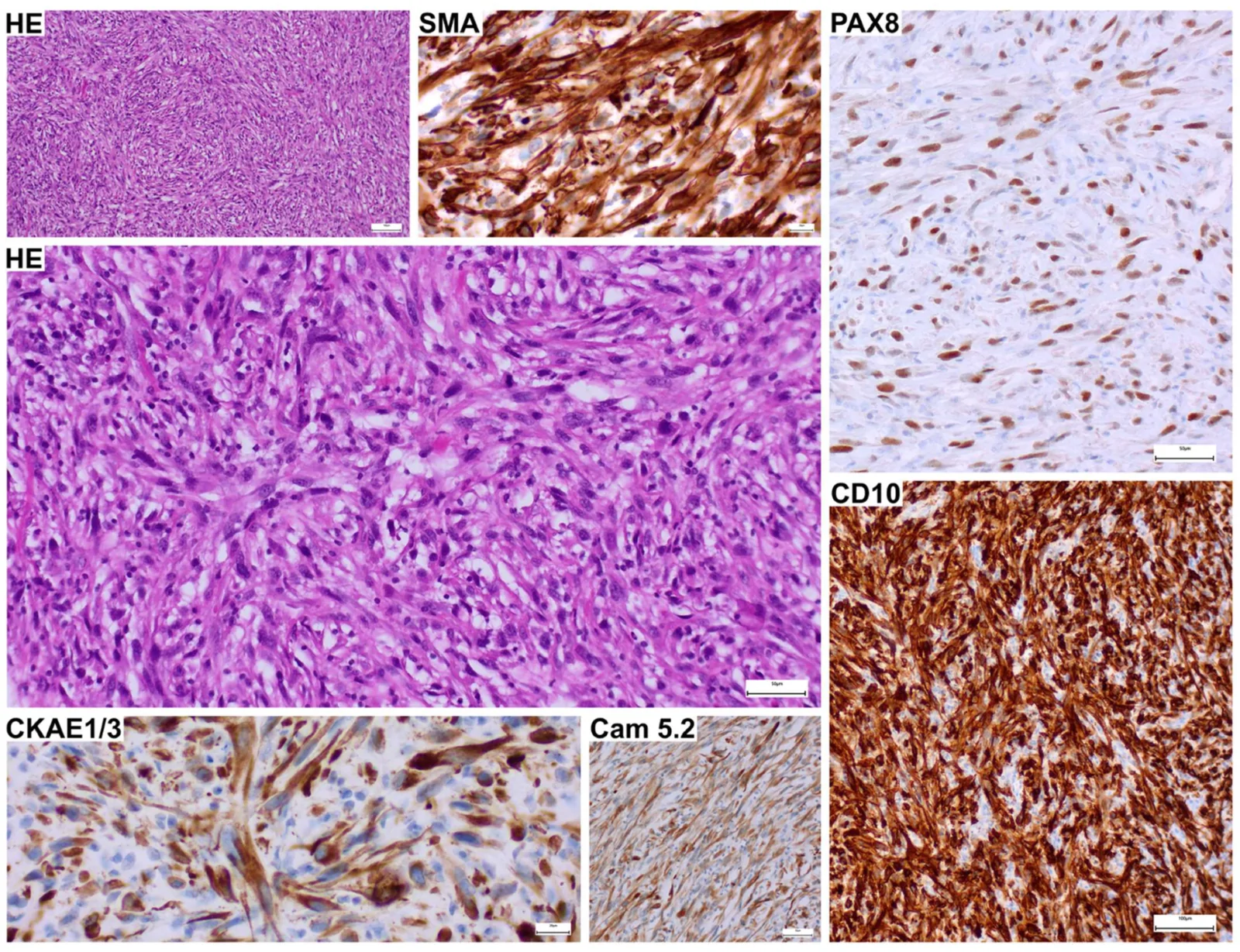
Sarcomatoid differentiation in renal cell carcinoma.

In biopsy material, subclassification of RCC with sarcomatoid differentiation may be limited or impossible, particularly when the sampled tissue consists predominantly of high-grade spindle cell or pleomorphic tumour (Figure 1).

Representative histological and immunohistochemical features of renal cell carcinoma with sarcomatoid differentiation include the following. Hematoxylin and eosin-stained sections show a high-grade spindle cell neoplasm arranged in intersecting fascicles and storiform areas, with marked nuclear atypia and loss of conventional epithelial architecture. Immunohistochemistry supports renal epithelial differentiation: the tumour cells express cytokeratins AE1/AE3 and CAM5.2, are PAX8-positive in the nucleus, show strong CD10 expression, and exhibit weak CAIX staining. SMA is positive in spindle tumour cells, highlighting myoid/myofibroblastic differentiation; however, desmin and h-caldesmon are negative, making primary smooth muscle sarcoma less likely. Additional negative markers include RCC marker, CK5/6, p63, cathepsin K, EMA, ALK1, CD34, SOX10, S100, Melan-A, and MDM2. This immunophenotype helps exclude sarcomatoid urothelial carcinoma, leiomyosarcoma, PEComa/epithelioid angiomyolipoma, melanoma, vascular neoplasm, and dedifferentiated liposarcoma. The combined morphology and immunoprofile are consistent with sarcomatoid differentiation of renal cell carcinoma.

In such cases, the underlying RCC subtype should not be assigned unless a conventional component or sufficiently specific immunophenotypic pattern is present. The diagnosis should acknowledge sarcomatoid differentiation and clearly state that further subclassification is not possible with the available biopsy material. This is especially relevant in patients who are not candidates for surgery in whom a biopsy may remain the only available tissue sample for pathological assessment. In this setting, immunohistochemistry is mainly used to support a renal epithelial origin and to exclude major mimics rather than providing precise RCC subtyping. PAX8, cytokeratins, and EMA may support a diagnosis of RCC. At the same time, additional markers should be selected based on the differential diagnosis, including sarcomatoid urothelial carcinoma, primary renal sarcoma, dedifferentiated retroperitoneal liposarcoma, PEComa/epithelioid angiomyolipoma, and metastatic melanoma. The proportion of sarcomatoid morphology in the biopsy may be described. However, it should be interpreted as a feature of the sampled tissue rather than a reliable estimate of the entire tumour.

## 4. Prognosis

Prognosis in sRCC is poor, with a median overall survival (OS) in patients with metastatic disease typically ranging from 5.9 to 13.3 months [18,19,37,47,48] with available reports of significantly longer OS [49]. Distant metastasis-free survival (DMFS) is often short, particularly in tumours with >30–50% sarcomatoid composition [50]. In one study of 98 patients presenting without metastases (M0/Mx), cancer-specific survival rates were 50% (95% CI: 41–58) at 2 years and 32% (95% CI: 24–41) at 5 years [3].

Retrospective analyses demonstrate that the extent of sarcomatoid differentiation is associated with progressively worse outcomes [9,51]. Patients with ≥25% sarcomatoid features have a 2.07-fold higher risk of overall mortality (*p* = 0.048), while those with ≥30% features have a 1.52-fold higher risk of RCC-specific mortality (*p* = 0.018) [52]. Each 10% increase in the sarcomatoid component is associated with a 6% increase in the risk of RCC-related death (*p* = 0.028) [52]. Stage-for-stage comparisons reveal cancer-specific mortality rate ratios of 1.4, 0.9, and 0.8 for stages I–II, III, and IV, respectively. Even after complete surgical resection, most non-metastatic sRCCs recur within two years. This is in stark contrast to the >90% five-year recurrence-free survival rate seen in non-sarcomatoid RCC [9].

## 5. Molecular Pathogenesis—Brief Overview

**Figure 2:**
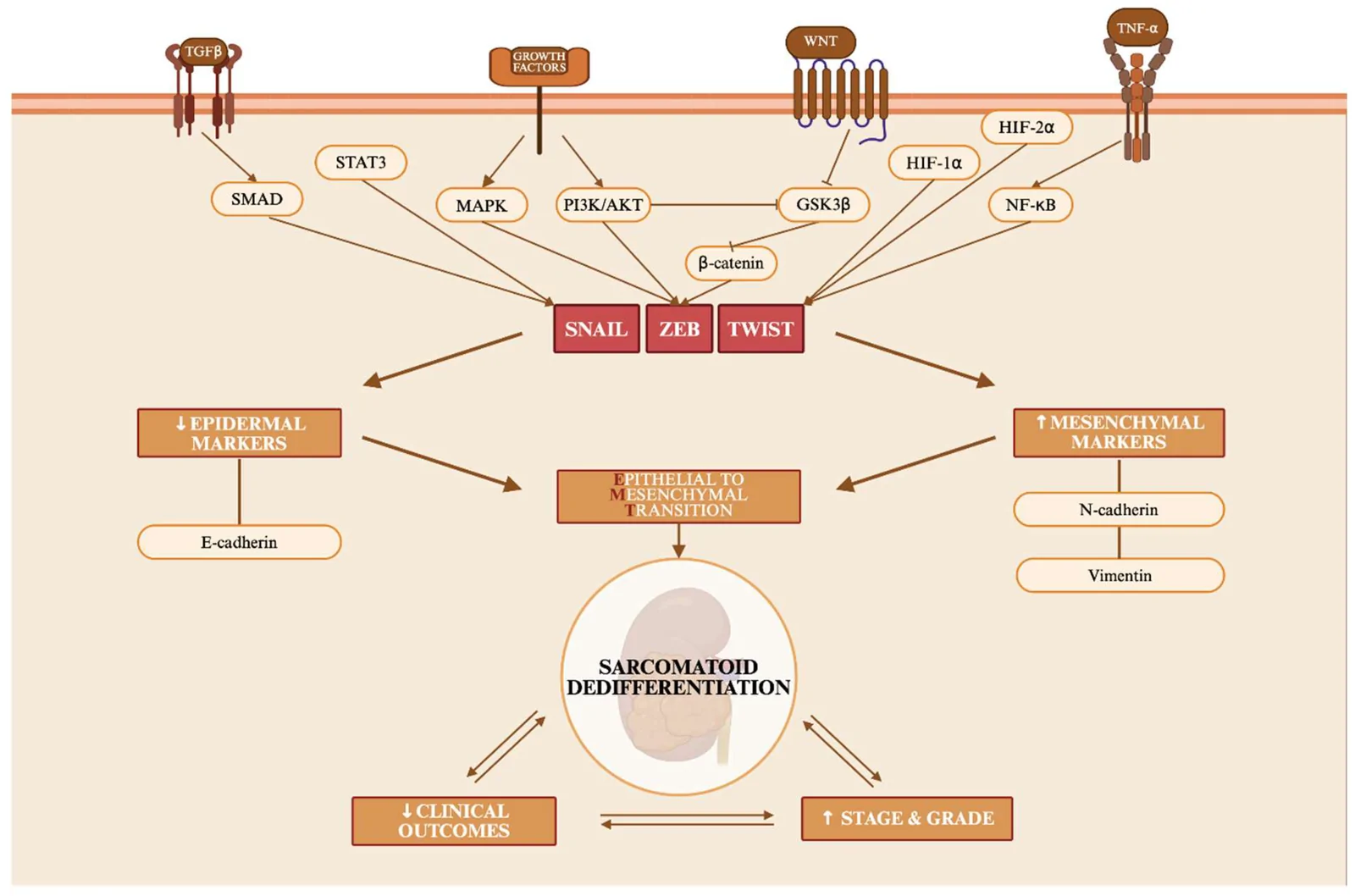
Molecular factors contributing to epithelial-to-mesenchymal transition and sarcomatoid dedifferentiation of RCC.

At the molecular level, sRCCs often harbour genomic alterations shared with their associated RCC component [27,53,54,55]. However, they also exhibit specific alterations that are enriched in the sarcomatoid component (Figure 2). This suggests that clonal evolution occurs as sarcomatoid features develop. Among the key genomic alterations enriched in the sarcomatoid component were *BAP1* mutations (33%), *CDKN2A/B* deletions (26% and 24%, respectively), and *NF2* mutations (6%), while *KDM5C* mutations were less common (4%) [55]. High-level amplifications in *MYC* (14%), EZH2 (13%), and CCNE1 (10%) were also observed, indicating dysregulated proliferation and chromatin remodelling. Transcriptomic analysis revealed consistent upregulation of *MYC* targets and pathways related to EMT, the cell cycle, and apoptosis, correlating with poor prognosis. High *MYC* transcriptional activity within sRCC was independently associated with worse OS in one genomic cohort [55].

Despite its aggressive nature, sRCC exhibited an immune-inflamed phenotype characterised by elevated CD8+ T cell infiltration, M1 macrophage polarisation, Th1 signalling, and PD-L1 expression on tumour cells (43%), independently of CD274 amplification. These features may, at least in part, underpin the heightened sensitivity of sRCC to ICIs, though causal relationships remain to be established in prospective studies. Molecular subtyping from CheckMate 214 identified an angiogenesis-low/T-effector-high tumour microenvironment subset—in which sarcomatoid tumours are enriched—as the group most responsive to nivolumab plus ipilimumab [55,56]. Notably, Bi et al. provided direct evidence of a shared clonal origin between the carcinomatous and sarcomatoid components of ccRCC, with 42% of somatic mutations being shared between the two [57]. The study demonstrated that sarcomatoid regions harbour a significantly higher mutation burden, particularly in cancer driver genes, and exhibit greater loss of heterozygosity. Notably, the study identified biallelic TP53 mutations as sarcomatoid-specific in 32% of cases. These mutations were absent in carcinomatous regions and mutually exclusive with BAP1 and ARID1A mutations, which were restricted to sarcomatoid areas.

## 6. Treatment of Localised Disease

### 6.1. Surgery

Surgical treatment of localised sRCC can offer a potential cure. Recurrence rates are high, approaching 80% within two years; this contrasts markedly with non-sarcomatoid RCC, where the 5-year recurrence-free survival rate after surgery is greater than 90% [58,59,60]. In one study, the 10-year RFS rates for T1a, T1b, and T2 RCC were 94.5%, 75.0%, and 57.9%, respectively [59].

The rate of partial nephrectomy (PN) for sRCC is generally low, particularly at advanced stages, with a decline in use as the disease progresses [59]. For instance, in one study, PN was performed in 30% of stage I, 5.4% of stage II, 4.4% of stage III, and 1.9% of stage IV sRCC cases [61]. In the same study, PN was independently associated with a 39% reduction in the hazard of all-cause mortality compared with RN (adjusted HR 0.61; 95% CI 0.49–0.76; *p* < 0.001) [61]. These findings are consistent with the propensity-score–matched analysis by Bhindi et al. [62], which included 452 patients with T1N0M0 sarcomatoid RCC (155 patients underwent PN; 297 underwent RN). The median follow-up period was 38 months (range 2–132) for PN and 39 months (range 0–150) for RN. Following propensity-score matching to balance confounding variables such as age, sex, and marital status, along with tumour size, the median overall survival was 132 months for patients receiving PN, compared with 100 months for those undergoing RN (*p* = 0.11). The median cancer-specific survival had not been reached in either group (*p* = 0.092) [62]. In general, observational evidence indicates that for T1 Tumours under 7 cm, PN achieves oncologic outcomes comparable to, if not better than, those of RN [63]. Notably, as mentioned, sRCCs characteristically present as large lesions (median diameter of approximately 10 cm) and typically display aggressive clinical behaviour [63,64]. Therefore, an RN is often necessary to achieve complete tumour resection, especially when the tumour is large and centrally located [21,65,66].

While RN was the standard approach for RCC in the past, with the advent of PN for smaller tumours and the focus on lymph node status in other urologic cancers, its role in larger tumours and sRCC remains under evaluation, with some studies questioning the benefit of cytoreductive nephrectomy in general [51]. Therefore, lymphadenectomy during nephrectomy is often recommended for patients with high-risk features. In a study by Merrill et al., a cohort of 77 patients with clinically localised sRCC, 2-Year OS was 50%; median RFS was 26.2 months. Node positivity independently predicted worse OS (HR 2.0, *p* < 0.001) and RFS (HR 2.1, *p* < 0.001). Findings support LND for staging and prognostication [50].

### 6.2. Perioperative Systemic Therapy

The perioperative management of sRCC remains a significant challenge due to its poor response to traditional cytotoxic and radiation therapies. Cytotoxic chemotherapy, whether used alone or in combination with targeted agents, remains ineffective in sRCC. In the phase II ECOG-8802 trial, which investigated the use of doxorubicin (50 mg/m^2^) plus gemcitabine (1500 mg/m^2^ on days 1 and 8) in patients with sarcomatoid features (*n* = 37 evaluable), the objective response rate was 16% (6/37; five partial responses and one complete response), with stable disease observed in 26% of patients (10/37) [67]. Median PFS was 3.5 months, and median OS was 8.8 months. Subsequent studies have failed to consistently replicate these outcomes [68,69,70].

Data on neoadjuvant and adjuvant ICIs in sRCC are limited due to small subgroup sizes. Nevertheless, both KEYNOTE-564 (NCT03142334) and IMmotion010 (NCT03024996) suggested improved disease-free survival and revealed a significant advantage in the sarcomatoid subgroup for KEYNOTE-564 (HR 0.29, 95% CI 0.09–0.91) [71,72]. Pembrolizumab has been approved as adjuvant therapy for patients with high-risk localised RCC, including sarcomatoid variants, based on the KEYNOTE-564 trial, in which 994 patients with high-risk non-metastatic clear cell RCC after nephrectomy (with or without prior metastasectomy) were randomised 1:1 to receive adjuvant pembrolizumab (200 mg intravenously every three weeks for up to 17 cycles) or placebo. At a median follow-up of 24.1 months, pembrolizumab was found to significantly prolong investigator-assessed disease-free survival (24-month DFS: 77.3% vs. 68.1%; HR for recurrence or death: 0.68; 95% CI: 0.53–0.87; *p* = 0.002), and demonstrated a favourable overall survival trend at 24 months (96.6% vs. 93.5%; HR for death: 0.54; 95% CI: 0.30–0.96). Grade 3 or higher adverse events occurred in 32.4% of patients treated with pembrolizumab versus 17.7% of those treated with placebo, and no treatment-related deaths were reported [73]. Updated OS data from KEYNOTE-564 (median follow-up 57.2 months) confirmed a statistically significant OS benefit: 48-month OS rates were 91.2% versus 86.0% (HR 0.62; 95% CI 0.44–0.87; *p* = 0.0024) [74]. The sarcomatoid subgroup (*n* = 111) showed the largest DFS benefit (HR 0.29, 95% CI 0.09–0.91) [71,74].

Moreover, in a meta-analysis of four randomised phase III trials of perioperative or adjuvant immune-checkpoint inhibition (KEYNOTE-564, IMmotion010, PROSPER [perioperative nivolumab, which did not meet its primary DFS endpoint in the overall population] and CheckMate914), which enrolled patients with high-risk resected renal cell carcinoma, 5–17% of participants in each study harboured sarcomatoid differentiation [73]. When these sarcomatoid subsets were pooled, adjuvant checkpoint blockade conferred a clear disease-free survival advantage (hazard ratio 0.59, 95% CI 0.38–0.91; I^2^ = 15%), despite divergent study designs and drug regimens (anti-PD-1, anti-PD-L1 and combination anti-CTLA-4/PD-1). This contrasted with the non-significant overall DFS benefit across (HR 0.85, 95% CI 0.69–1.04; I^2^ = 64%), highlighting the sensitivity of sarcomatoid tumours to immunomodulation. The sarcomatoid subgroup experienced toxicity similar to that observed in broader cohorts, with an increased incidence of Grade ≥3 adverse events (OR 2.51), immune-related toxicities (OR 4.79), and treatment discontinuation (OR 10.63) in the ICI arms [73].

### 6.3. Perioperative Radiotherapy

The SEER analysis, specifically in non-metastatic sRCC, showed no improvement in DSS or OS [21,75,76]. A total of 408 non-metastatic sRCC patients who received any surgical treatment (PN or RN, with or without ureterectomy/bladder cuff, or RN with adjuvant external-beam radiation) exhibited significantly higher 5-year OS (20.1–54.0% vs. 9.0% in the no-treatment group; *p* < 0.001) and DSS (20.1–59.9% vs. 11.6%; *p* < 0.001). Surgical intervention was independently associated with reduced cause-specific mortality in a multivariable competing-risks analysis (*p* < 0.0001). However, there were no significant differences in OS or in 1-, 3- or 5-year DSS between RN alone and RN with adjuvant radiation. Adjuvant radiation did not decrease 1-year cause-specific mortality (HR 0.95; 95% CI 0.23–3.96; *p* = 0.947), indicating no survival benefit of postoperative radiotherapy in non-metastatic sRCC [77,78].

## 7. Treatment of Unresectable/Metastatic Disease

### 7.1. Surgery

sRCC is associated with a high risk of developing metastases. Therefore, in most cases, treatment is palliative. Upfront cytoreductive nephrectomy is generally not recommended in patients with sRCC, as existing data indicate limited clinical benefit and poor outcomes with this approach [79]. However, metastasectomy is a treatment option, particularly for patients with a limited number of metastases (oligometastatic disease) and a favourable performance status. However, the data are scarce and often inconclusive [79,80,81,82,83]. For instance, in one study of 40 patients who underwent metastasectomy, complete resection was targeted at bone (57% synchronous and 10% asynchronous), the brain (30% synchronous and 50% asynchronous), the lung (7% synchronous and 20% asynchronous), and intra-abdominal and other sites [82,83]. Two synchronous patients underwent multi-site resection at their first surgery. Of the synchronous patients 63% and of the asynchronous patients 50% required only one metastasectomy session (up to four sessions were performed). However, metastasectomy did not significantly improve survival: median OS was 8.4 vs. 8.0 months in synchronous disease (*p* = 0.35) and 36.2 vs. 13.7 months in asynchronous metastases (*p* = 0.29) [84]. In general, metastasectomy in oligometastatic RCC has been associated with the greatest benefit for patients under 60 years of age with a disease-free interval exceeding one year or solitary pulmonary metastases [85,86]. However, evidence for the efficacy of metastasectomy in the sarcomatoid variant is scarce and confined to small retrospective case series, leaving its true impact unresolved [79,80,81,82,83,87].

### 7.2. Palliative Systemic Therapy

Active surveillance is usually only recommended for patients with favourable-risk metastatic RCC who are asymptomatic and have a low disease burden [9,88]. However, this approach is not appropriate for patients with sRCC, as these tumours are characterised by aggressive behaviour and rapid progression. They are also unlikely to meet the clinical criteria necessary for safe observation without immediate systemic therapy. Data on active surveillance in metastatic sRCC are largely limited to subgroup and observational analyses; sarcomatoid dedifferentiation does not, per se, dictate a poorer response to systemic therapy (Table 2). For instance, in a retrospective real-world analysis of 173 patients with metastatic clear cell RCC, first-line ipilimumab + nivolumab (IO/IO; *n* = 90, 31 per cent sarcomatoid) produced a median PFS of 6.8 months (95 per cent CI 4.5–12.2), whereas VEGFR-TKI + PD-1 combinations (TKI/IO; *n* = 83, 20 per cent sarcomatoid) achieved a median PFS of 21 months (95 per cent CI 15–25; *p* = 0.009) [89]. At 48 months, adjusted RMST for PFS was 10 months with IO/IO versus 24 months with TKI/IO (*p* = 0.02), while RMST for second-line PFS was 20 months versus 23 months, respectively (*p* = 0.24). There was no significant interaction between sarcomatoid status and treatment effect for either PFS (*p* = 0.95) or PFS-2 (*p* = 0.29), indicating superior first-line disease control with TKI/IO but similar downstream efficacy regardless of sarcomatoid dedifferentiation [89].

The combination of nivolumab and ipilimumab is the preferred first-line therapy for the treatment of metastatic clear cell RCC with sarcomatoid features in patients who have not received prior treatment [90]. However, real-world comparative analyses suggest that TKI/IO combinations (e.g., pembrolizumab plus axitinib) may achieve superior median PFS over IO/IO doublets in sarcomatoid ccRCC (21 vs. 6.8 months; *p* = 0.009) [89]; regimen selection should therefore be individualised according to IMDC risk category, disease burden, and the need for rapid cytoreduction [89,90]. This regimen has been shown to deliver durable, complete responses and long-term OS and treatment-free survival, outperforming sunitinib [91]. FDA approval is based on IMDC intermediate- and poor-risk criteria, which encompass most sRCC patients. However, given its efficacy, this regimen is also offered to favourable-risk patients with sarcomatoid histology [90]. In the primary sarcomatoid subgroup analysis of CheckMate 214 (NCT02231749; *n* = 139), nivolumab plus ipilimumab significantly improved OS, PFS, and ORR compared to sunitinib, regardless of PD-L1 expression [90]. After 5 years of follow-up, median OS had not been reached with the combination, compared with 14.2 months with sunitinib (HR 0.46); the 5-year OS rates were 47% versus 21%, respectively [90]. Median PFS was 27 months versus six months (HR 0.50), and the five-year PFS rate was 46% versus 12%. The ORR was 61% with the ICI combination versus 23% with sunitinib, with complete responses in 23% and 6% of patients, respectively. In a prespecified subgroup analysis of patients with sarcomatoid features (phase III KEYNOTE-426 trial; NCT02853331), pembrolizumab plus axitinib significantly improved OS (HR 0.73, 95% CI 0.60–0.88) and PFS (HR 0.69, 95% CI 0.59–0.81) versus sunitinib across all risk groups (*n* = 861) [90,92]. A prespecified subgroup analysis of patients with sarcomatoid features (pembro + axi vs. sunitinib) similarly demonstrated pronounced activity in the sarcomatoid subgroup, with an ORR of 58.8% (vs. 31.5% with sunitinib), a CR rate of 11.8% versus 0%, and tumour shrinkage in 98% of patients—outcomes significantly superior to those seen in the overall RCC population [90,92]. Moreover, the activity of single-agent pembrolizumab in sRCC was evaluated in the phase II KEYNOTE-427 trial (NCT02853344), which comprised two cohorts [91,93]. Cohort A comprised treatment-naïve clear cell RCC patients, 10% (11/110) of whom had sarcomatoid features. Cohort B included non-clear cell RCC patients where 23% (38/165) had an sRCC diagnosis. At a median follow-up of 32 months, the objective response and complete response rates among sarcomatoid patients in Cohort B were 42% and 8%, respectively [91,93].

In contrast, combination regimens involving ICIs and tyrosine kinase inhibitors (TKIs) have demonstrated notable clinical activity in patients with sRCC [94,95]. The CheckMate 9ER trial (nivolumab plus cabozantinib) demonstrated superior PFS versus sunitinib (HR 0.51, 95% CI 0.41–0.64) [96]. Similarly, the CLEAR trial (KEYNOTE-581; NCT02811861) evaluated lenvatinib plus pembrolizumab, reporting a median PFS of 23.9 months and an ORR of up to 71% in the overall cohort. In a subgroup analysis, patients with sarcomatoid features derived benefit consistent with the overall population [97]. In a subgroup analysis, patients with sarcomatoid features derived a benefit consistent with the overall population. Avelumab plus axitinib (A + Ax) targets PD-L1 and VEGFR signalling. In the JAVELIN Renal 101 sRCC post hoc cohort analysis (whole cohort: *n* = 108; A + Ax: *n* = 47; sunitinib: *n* = 61), A + Ax improved median PFS versus sunitinib [stratified HR: 0.57 (95% confidence interval, 0.325–1.003] [95,98]. Moreover, atezolizumab plus bevacizumab (Atezo + Bev) also targets PD-L1 and VEGF signalling. In the prespecified subgroup analysis of the IMmotion 151 trial (*n* = 142; Atezo + Bev, *n* = 68; sunitinib, *n* = 74), Atezo + Bev improved PFS (8.3 vs. 5.3 months [HR 0.52, 95% CI 0.34–0.79]) and increased ORR (49% vs. 14% (CR 10% vs. 3%)) with similar safety to the overall study [99,100]. OS was neutral in the intent-to-treat population at final analysis, so use should be individualised, typically when rapid cytoreduction is required and PD-L1 positivity is present [38]. Importantly, for patients with non-clear cell RCC subtypes harbouring sarcomatoid features, the phase III SUNNIFORECAST trial (NCT03075423) represents the first randomised study demonstrating the superiority of nivolumab plus ipilimumab over standard care, with a 12-month OS rate of 86.9% versus 76.8% (*p* = 0.014) and a median OS of 42.4 versus 33.9 months, particularly in high-risk papillary RCC [45]. These data provide a formal evidence base for the use of dual ICI therapy across non-ccRCC histologies with sarcomatoid differentiation.

### 7.3. Palliative Radiotherapy

In terms of limitations of the evidence base for non-clear cell sRCC, the majority of clinical trial data on systemic therapy in sRCC are derived from subgroup analyses of trials designed for clear cell RCC; dedicated prospective studies are largely absent [81]. For patients with non-ccRCC harbouring sarcomatoid differentiation (papillary, chromophobe, unclassified), evidence is particularly sparse, limited mainly to the SUNNIFORECAST trial [101] and small retrospective series. Response rates and OS in non-ccRCC sRCC may differ substantially from ccRCC sRCC; extrapolation of treatment recommendations across subtypes should be made with caution. ESMO [102] and EAU [103] guidelines acknowledge this evidence gap and recommend enrolling patients in clinical trials wherever possible.

High-dose stereotactic radiosurgery (SRS) achieves a 75% response rate in RCC brain metastases, and aggressive stereotactic body radiotherapy (SBRT) regimens—typically ≥45 Gy (up to 48 Gy delivered in 3–4 fractions)—provide effective local tumour control. Therefore, radiotherapy for sRCC is primarily used to treat symptomatic metastatic lesions palliatively, with high-dose SRS/SBRT [81]. Spine-specific cohorts demonstrate high LC and meaningful analgesia; pain improvement ranges from 41% to 95%. SBRT shows superior LC compared with conventional RT, albeit with a risk of vertebral fractures, requiring patient selection [76,77,81].

**Table 2:** Systemic therapies in sarcomatoid renal cell carcinoma: mechanisms, indications, trial identifiers, and key outcomes in sarcomatoid subsets.

Drug/Regimen	Class	Molecular Target/Pathway	Approved/Typical Use in RCC	Trial (ClinicalTrials.gov Registration Number(s))	N (sRCC/RCC (%sRCC))	Results
Nivolumab + ipilimumab	ICI + ICI	*PD-1 + CTLA-4*	1L advanced RCC, particularly IMDC intermediate/poor risk	CheckMate-214 (NCT02231749)	145/1096 (13.2%)	sRCC subgroup (*n* = 139, intermediate/poor-risk): ORR 61% vs. 23%; CR 23% vs. 6%; 5 yr OS 47% vs. 21% (HR 0.46); median PFS 27 vs. 6 mo (HR 0.50). 9 yr follow-up: 30% alive vs. 18% (sunitinib), 21% progression-free; half of responders in ongoing remission without further therapy [91,101,104,105].
Pembrolizumab + axitinib	ICI + TKI	*PD-1 + VEGFR1-3*	1L RCC (all risk groups)	KEYNOTE-426 final (NCT02853331)	104/861 (12.1%)	Overall: OS HR 0.73 (95% CI 0.60–0.88); PFS HR 0.69 (0.59–0.81); ORR 60.6% vs. 39.6% vs. sunitinib. sRCC subgroup: ORR 58.8% vs. 31.5%; CR 11.8% vs. 0%; tumour shrinkage in 98% [90,92,96,106].
Nivolumab + cabozantinib	ICI + TKI	*PD-1 + VEGFR2/MET/AXL*	1L advanced RCC (all risk groups)	CheckMate-9ER final (NCT03141177)	75/651 (11.5%)	PFS: HR 0.58; OS: HR 0.79 (0.65–0.96) at ~33 months; ORR 55.9% vs. 28% vs. sunitinib; CR 13.9% vs. 4.6% [107].
Lenvatinib + pembrolizumab	ICI + TKI	*PD-1 + VEGFR1-3/FGFR1-4/PDGFRα/RET/KIT*	1L advanced RCC (all risk groups)	CLEAR/KEYNOTE-581 (NCT02811861)	28/355 (7.9%)	mPFS 23.9 vs. 9.2 mo, HR 0.39 (95% CI 0.32–0.49); OS benefit significant; ORR 71.3% vs. 36.7% vs. sunitinib [97].
Avelumab + axitinib	ICI + TKI	*PD-L1 + VEGFR1-3*	1L advanced RCC (some regions)	JAVELIN Renal 101 (NCT02684006)	108/886 (12.2%)	PFS benefit vs. sunitinib; OS data considered immature even in updated analyses [95,98,108].
Atezolizumab + bevacizumab	ICI + anti-VEGF mAb	*PD-L1 + VEGF-A*	1L RCC in select settings	IMmotion151 (NCT02420821)	142/915 (15.5–16%)	PFS benefit in the PD-L1+ subgroup; no OS advantage vs. sunitinib.
Pembrolizumab (monotherapy)	ICI, adjuvant	*PD-1*	Adjuvant after nephrectomy for ccRCC is at increased risk of LR	KEYNOTE-564 (NCT03142334)	111/994 (11.2%)	DFS HR 0.72–0.68 across reports; OS benefit significant at 57 mo follow-up with HR 0.62 (0.44–0.87).
Sunitinib	TKI	*VEGFR1-3/PDGFR/KIT/FLT3/RET*	Historic 1L standard; benchmark comparator	Sunitinib vs. IFN-α (NCT00083889; NCT00098657)	NR	mPFS 11 vs. 5 mo; ORR 31% vs. 6%.
Pazopanib	TKI	*VEGFR1-3/PDGFR/KIT*	1L alternative to sunitinib	COMPARZ (NCT00720941)	NR	Non-inferior efficacy to sunitinib with a different toxicity profile;
Axitinib	TKI	*VEGFR1-3* (selective)	2L after prior systemic therapy	AXIS (NCT00678392)	NR	mPFS 6.7 vs. 4.7 mo vs. sorafenib, HR 0.665 (0.544–0.812).
Cabozantinib	TKI	*VEGFR2/MET/AXL*	2L after *VEGF*-targeted therapy; also 1L in combination	METEOR (NCT01865747)	NR	OS HR 0.66 (0.53–0.83) vs. everolimus; PFS 7.4 vs. 3.8 mo.
Tivozanib	TKI	*VEGFR1-3* (selective)	3L+ after ≥2 prior regimens	TIVO-3 (NCT02627963)	NR	mPFS 5.6 vs. 3.9 months vs. sorafenib; HR 0.73 (0.56–0.94); favourable tolerability.
Lenvatinib + everolimus	TKI + mTOR	*VEGFR/FGFR + mTORC1*	Post-VEGF-TKI setting	LEN + EVE Phase II (NCT01136733)	NR	mPFS 14.6 vs. 5.5 mo vs. everolimus, HR 0.40 (95% CI 0.24–0.68); ORR 43% vs. 6%.
Everolimus	mTOR inhibitor	*mTORC1*	Post-VEGFR-TKI progression	RECORD-1 (NCT00410124)	NR	mPFS 4.9 vs. 1.9 mo vs. placebo; HR 0.33.
Temsirolimus	mTOR inhibitor	*mTORC1*	1L for poor-risk mRCC	Global ARCC (NCT00065468)	NR	OS HR 0.73 (0.58–0.92) vs. IFN-α; mOS 10.9 vs. 7.3 mo.
Belzutifan	HIF-2α inhibitor	*HIF-2α* transcription factor	Previously treated ccRCC after ICI and anti-angiogenic therapy	LITESPARK-005(NCT04195750)	NR	ORR 21.9% vs. 3.5%; 18 mo PFS 24.0% vs. 8.3% vs. everolimus; OS HR 0.88 (ns).
Bevacizumab + IFN-α	Anti-VEGF mAb	*VEGF-A ligand*	1L option in select regions	AVOREN(NCT00738530)	NR	Improved PFS and ORR vs. IFN-α alone; OS not significantly different.
Sunitinib (adjuvant)	TKI, adjuvant	*VEGFR1-3/PDGFR/KIT/FLT3/RET*	High-risk post-nephrectomy—DFS benefit only; toxicity limits use	S-TRAC(NCT00375674)	NR	DFS HR 0.76 (0.59–0.98) vs. placebo; no OS benefit; higher grade 3–4 toxicity.

Notes: NR—*not reported* means sarcomatoid differentiation was not prospectively captured or reported in the pivotal publication; any sRCC data for this regimen are derived from post hoc, non-pivotal, or real-world analyses. Abbreviations: RCC—renal cell carcinoma; ccRCC—clear cell RCC; IMDC—International Metastatic RCC Database Consortium; ICI—immune checkpoint inhibitor; TKI—tyrosine-kinase inhibitor; mAb—monoclonal antibody; PD-1/PD-L1/CTLA-4—programmed cell death-1/ligand-1/cytotoxic T-lymphocyte antigen-4; VEGFR—vascular endothelial growth factor receptor; mTORC1—mechanistic target of rapamycin complex-1; HIF-2α—hypoxia-inducible factor-2α; OS—overall survival; PFS—progression-free survival; DFS—disease-free survival; ORR—objective response rate; CR—complete response.

## 8. Summary and Conclusions

sRCC is a clinically aggressive and molecularly distinct tumour [2]. It is prevalent in advanced stages and associated with a significantly worse prognosis than non-sarcomatoid RCC [109]. Traditional therapies, including cytotoxic chemotherapy, radiation therapy, and surgery alone, have a limited impact on long-term survival. Recent insights into the molecular landscape of sRCC have revealed recurrent alterations in TP53, BAP1, and CDKN2A/B, as well as a highly inflamed tumour microenvironment characterised by PD-L1 expression and immune cell infiltration [1,53,54]. These features underpin the robust efficacy of ICIs, which have now become central to the treatment paradigm. In both metastatic and high-risk localised settings, ICI and/or TKI combination regimens significantly improve OS and ORR, with 9-year follow-up from CheckMate 214 (NCT02231749) now establishing durable remission in approximately 30% of intermediate- and poor-risk patients [46]. Despite these advances, outcomes remain suboptimal, and recurrence is common, even after complete resection. Cytoreductive nephrectomy and metastasectomy may be applied selectively, but systemic therapy is fundamental [102]. Future research should focus on refining risk stratification, validating predictive biomarkers, and evaluating neoadjuvant and adjuvant immunotherapy in prospective trials, and sRCC should be managed as a distinct clinical and molecular entity within the RCC spectrum.

## Figures and Tables

**Table 1:** Differential diagnosis and supportive role of immunohistochemistry in sarcomatoid renal cell carcinoma.

Diagnostic Problem	Useful IHC Panel	Expected/Helpful Pattern	Main Interpretative Points
Confirming renal epithelial origin in a spindle cell tumour	PAX8, AE1/AE3, CAM5.2, EMA, CAIX, CD10, RCC marker, vimentin	Sarcomatoid RCC often retains at least focal expression of epithelial markers; PAX8, CAIX, and CD10 may support renal origin.	Loss or focality of keratin/EMA is common in dedifferentiated areas. A negative single epithelial marker does not exclude sRCC. PAX8 is particularly helpful but may be reduced or heterogeneous. PAX8, CAIX, and CD10 are useful for confirming renal origin in sarcomatoid cells [14,30,31].
Clear cell RCC with sarcomatoid differentiation	PAX8, CAIX, CD10, vimentin, CK7, AMACR	CAIX typically diffuses in a membranous pattern in conventional clear cell areas; CD10 and vimentin are often positive; CK7 is usually negative or focal.	In the sarcomatoid component, CAIX/CD10 may be retained or lost. Search carefully for conventional clear cell areas, especially at the tumour periphery or near necrotic zones [30,32].
Papillary RCC with sarcomatoid differentiation	PAX8, CK7, AMACR, CD10, CAIX	CK7 and AMACR are often positive; CAIX lacks the classic diffuse box-like pattern of clear cell RCC.	Papillary architecture may be overgrown by spindle cell dedifferentiation. Foamy macrophages, psammoma bodies, or residual papillary areas may help [33,34,35]
Chromophobe RCC with sarcomatoid differentiation	PAX8, CK7, CD117/KIT, E-cadherin, Hale colloidal iron if used locally	Conventional areas are often CK7- and CD117-positive.	Sarcomatoid change may be extensive. Look for residual chromophobe morphology: plant–cell borders, perinuclear halos, raisinoid nuclei [33,34,35].
Collecting duct carcinoma/renal medullary carcinoma with sarcomatoid or undifferentiated morphology	PAX8, CK7, HMWCK, p63/p40, GATA3, INI1/SMARCB1	Collecting duct carcinoma is often CK7/HMWCK-positive; renal medullary carcinoma shows loss of INI1/SMARCB1.	Location in medulla/sinus, desmoplastic stroma, infiltrative tubulopapillary, or ductal growth and clinical context are essential. INI1 loss strongly supports SMARCB1-deficient renal medullary carcinoma in the correct setting [32,33].
Sarcomatoid urothelial carcinoma involving renal pelvis/sinus	GATA3, p63, p40, uroplakin II/III, CK7, CK20, PAX8	Urothelial carcinoma usually GATA3/p63/p40 positive; PAX8 usually negative.	One of the most important mimics. Look for urothelial carcinoma in situ, renal pelvis epicentre, papillary urothelial component, or mucosal involvement. PAX8/GATA3 combination is useful in separating sarcomatoid RCC from sarcomatoid urothelial carcinoma [33,34,43].
Primary renal leiomyosarcoma/other renal sarcoma	SMA, desmin, h-caldesmon, AE1/AE3, EMA, PAX8	Leiomyosarcoma: SMA/desmin/h-caldesmon positive; keratin/PAX8 negative.	Sarcomatoid RCC may express SMA or exhibit myofibroblastic features, so muscle markers alone are insufficient. Any epithelial/PAX8 positivity or residual RCC component favours sarcomatoid RCC [3,11,34,35,37,44].
Dedifferentiated liposarcoma secondarily involving the kidney	MDM2, CDK4, p16, PAX8, cytokeratins	MDM2/CDK4 positive or amplified; PAX8 negative.	Consider when the mass is predominantly retroperitoneal with renal involvement rather than renal-centred. Sampling should search for well-differentiated liposarcoma [35,39,41]
Epithelioid angiomyolipoma/PEComa	HMB45, Melan-A, cathepsin K, SMA, desmin, PAX8, cytokeratins	PEComa: melanocytic markers positive, often SMA positive; PAX8 and epithelial markers negative.	It can be highly atypical, epithelioid, spindle cell, or necrotic. Lack of PAX8/keratin and strong melanocytic marker expression support the diagnosis of PEComa [28,31].
Metastatic melanoma	SOX10, S100, Melan-A, HMB45, PRAME, PAX8, cytokeratins	Melanoma: SOX10/S100 positive; PAX8 negative; keratins usually negative.	Important in pleomorphic or spindle cell tumours. Clinical history and multifocal disease pattern are critical [28,31].
Vascular sarcoma/angiosarcoma	ERG, CD31, CD34, FLI1, cytokeratins, PAX8	Angiosarcoma: ERG/CD31 positive; PAX8 negative.	Sarcomatoid RCC can be very haemorrhagic and necrotic, but true vasoformative channels and strong endothelial markers favour vascular sarcoma [28,31]
Post-treatment change/stromal reaction mimicking sarcomatoid growth	Broad epithelial/RCC panel guided by residual viable tumour	Reactive spindle cells lack overt malignant cytology and usually lack epithelial/RCC marker expression.	Fibrosis, granulation tissue, therapy-related atypia, necrosis with organisation, and inflammatory pseudotumour-like areas should not be overcalled as sarcomatoid differentiation without unequivocal malignant spindle/pleomorphic tumour cells [28,31].

## Data Availability

No new data were created or analysed in this study. Data sharing does not apply to this article.

## References

[1] Debien V., Thouvenin J., Lindner V., Barthélémy P., Lang H., Flippot R., Malouf G.G. (2019). Sarcomatoid Dedifferentiation in Renal Cell Carcinoma: From Novel Molecular Insights to New Clinical Opportunities. Cancers.

[2] Delahunt B., Srigley J.R., Egevad L., Montironi R. (2014). International Society of Urological Pathology Grading and Other Prognostic Factors for Renal Neoplasia. Eur. Urol..

[3] Adibi M., Thomas A.Z., Borregales L.D., Merrill M.M., Slack R.S., Chen H.-C., Sircar K., Murugan P., Tamboli P., Jonasch E. (2015). Percentage of sarcomatoid component as a prognostic indicator for survival in renal cell carcinoma with sarcomatoid dedifferentiation. Urol. Oncol. Semin. Orig. Investig..

[4] Keskin S.K., Msaouel P., Hess K.R., Yu K.-J., Matin S.F., Sircar K., Tamboli P., Jonasch E., Wood C.G., Karam J.A. (2017). Outcomes of Patients with Renal Cell Carcinoma and Sarcomatoid Dedifferentiation Treated with Nephrectomy and Systemic Therapies: Comparison between the Cytokine and Targeted Therapy Eras. J. Urol..

[5] Cangiano T., Liao J., Naitoh J., Dorey F., Figlin R., Belldegrun A. (1999). Sarcomatoid renal cell carcinoma: Biologic behavior, prognosis, and response to combined surgical resection and immunotherapy. J. Clin. Oncol..

[6] Hahn A.W., Lebenthal J., Genovese G., Sircar K., Tannir N.M., Msaouel P. (2022). The significance of sarcomatoid and rhabdoid dedifferentiation in renal cell carcinoma. Cancer Treat. Res. Commun..

[7] Pichler R., Compérat E., Klatte T., Pichler M., Loidl W., Lusuardi L., Schmidinger M. (2019). Renal Cell Carcinoma with Sarcomatoid Features: Finally New Therapeutic Hope?. Cancers.

[8] He H., Magi-Galluzzi C. (2014). Epithelial-to-Mesenchymal Transition in Renal Neoplasms. Adv. Anat. Pathol..

[9] Blum K.A., Gupta S., Tickoo S.K., Chan T.A., Russo P., Motzer R.J., Karam J.A., Hakimi A.A. (2020). Sarcomatoid renal cell carcinoma: Biology, natural history and management. Nat. Rev. Urol..

[10] Amin M.B., Tamboli P., Javidan J., Stricker H., Venturina M.D.-P., Deshpande A., Menon M. (2002). Prognostic Impact of Histologic Subtyping of Adult Renal Epithelial Neoplasms. Am. J. Surg. Pathol..

[11] de Peralta-Venturina M., Moch H., Amin M., Tamboli P., Hailemariam S., Mihatsch M., Javidan J., Stricker H., Ro J.Y., Amin M.B. (2001). Sarcomatoid Differentiation in Renal Cell Carcinoma. Am. J. Surg. Pathol..

[12] Cheville J.C., Lohse C.M., Zincke H., Weaver A.L., Leibovich B.C., Frank I., Blute M.L. (2004). Sarcomatoid renal cell carcinoma: An examination of underlying histologic subtype and an analysis of associations with patient outcome. Am. J. Surg. Pathol..

[13] Gu L., Li H., Wang H., Ma X., Wang L., Chen L., Zhao W., Zhang Y., Zhang X. (2016). Presence of sarcomatoid differentiation as a prognostic indicator for survival in surgically treated metastatic renal cell carcinoma. J. Cancer Res. Clin. Oncol..

[14] Alevizakos M., Gaitanidis A., Nasioudis D., Msaouel P., Appleman L.J. (2019). Sarcomatoid Renal Cell Carcinoma: Population-Based Study of 879 Patients. Clin. Genitourin. Cancer.

[15] Ro J.Y., Ayala A.G., Sella A., Samuels M.L., Swanson D.A. (1987). Sarcomatoid renal cell carcinoma: Clinicopathologic. A study of 42 cases. Cancer.

[16] Tomera K.M., Farrow G.M., Lieber M.M. (1983). Sarcomatoid Renal Carcinoma. J. Urol..

[17] Bertoni F., Ferri C., Benati A., Bacchini P., Corrado F. (1987). Sarcomatoid Carcinoma of the Kidney. J. Urol..

[18] Shuch B., Bratslavsky G., Linehan W.M., Srinivasan R. (2012). Sarcomatoid renal cell carcinoma: A comprehensive review of the biology and current treatment strategies. Oncologist.

[19] Chen X.-Y., Lan M., Zhou Y., Chen W.-Z., Hu D., Liu J.-M., Huang S.-H., Liu Z.-L., Zhang Z.-H. (2017). Risk factors for bone metastasis from renal cell cancer. J. Bone Oncol..

[20] Ullah A., Yasinzai A.Q.K., Sakhalkar O.V., Lee K.T., Khan I., Tareen B., Wali A., Waheed A., Khan J., Andam G. (2023). Demographic Patterns and Clinicopathological Analysis of Sarcomatoid Renal Cell Carcinoma in US Population. Clin. Genitourin. Cancer.

[21] Candelario N., Geiger C., Flaig T. (2021). Sarcomatoid Renal Cell Carcinoma: The Present and Future of Treatment Paradigms. Kidney Cancer.

[22] Park H.K. (2023). The Metastasis Pattern of Renal Cell Carcinoma Is Influenced by Histologic Subtype, Grade, and Sarcomatoid Differentiation. Medicina.

[23] Cheng M., Duzgol C., Kim T.-H., Ghafoor S., Becker A.S., Andrieu P.I.C., Gangai N., Jiang H., Hakimi A.A., Vargas H.A. (2023). Sarcomatoid renal cell carcinoma: MRI features and their association with survival. Cancer Imaging.

[24] Takeuchi M., Kawai T., Suzuki T., Naiki T., Kawai N., Fujiyoshi Y., Inagaki H., Kohri K., Hara M., Shibamoto Y. (2015). MRI for differentiation of renal cell carcinoma with sarcomatoid component from other renal tumor types. Abdom. Imaging.

[25] Abel E.J., Culp S.H., Matin S.F., Tamboli P., Wallace M.J., Jonasch E., Tannir N.M., Wood C.G. (2010). Percutaneous Biopsy of Primary Tumor in Metastatic Renal Cell Carcinoma to Predict High Risk Pathological Features: Comparison with Nephrectomy Assessment. J. Urol..

[26] Wei S., Al-Saleem T. (2017). The Pathology and Molecular Genetics of Sarcomatoid Renal Cell Carcinoma: A Mini-Review. J. Kidney Cancer VHL.

[27] Wang Z., Kim T.B., Peng B., Karam J., Creighton C., Joon A., Kawakami F., Trevisan P., Jonasch E., Chow C.-W. (2017). Sarcomatoid Renal Cell Carcinoma Has a Distinct Molecular Pathogenesis, Driver Mutation Profile, and Transcriptional Landscape. Clin. Cancer Res..

[28] Abel E.J., Heckman J.E., Hinshaw L., Best S., Lubner M., Jarrard D.F., Downs T.M., Nakada S.Y., Lee F.T., Huang W. (2015). Multi-Quadrant Biopsy Technique Improves Diagnostic Ability in Large Heterogeneous Renal Masses. J. Urol..

[29] Yu W., Wang Y., Jiang Y., Zhang W., Li Y. (2017). Distinct immunophenotypes and prognostic factors in renal cell carcinoma with sarcomatoid differentiation: A systematic study of 19 immunohistochemical markers in 42 cases. BMC Cancer.

[30] Anderson D.A., Tretiakova M. (2023). Sarcomatoid Renal Cell Carcinoma. https://www.pathologyoutlines.com/topic/kidneytumormalignantrccsarcoma.html.

[31] Delahunt B., Bethwaite P.B., McCredie M.R.E., Nacey J.N. (2007). The evolution of collagen expression in sarcomatoid renal cell carcinoma. Hum. Pathol..

[32] Albadine R., Schultz L., Illei P., Ertoy D., Hicks J., Sharma R., Epstein J.I., Netto G.J. (2010). PAX8 (+)/p63 (-) immunostaining pattern in renal collecting duct carcinoma (CDC): A useful immunoprofile in the differential diagnosis of CDC versus urothelial carcinoma of upper urinary tract. Am. J. Surg. Pathol..

[33] Gonzalez-Roibon N., Albadine R., Sharma R., Faraj S.F., Illei P.B., Argani P., Ertoy D., Allaf M.E., Netto G.J. (2013). The role of GATA binding protein 3 in the differential diagnosis of collecting duct and upper tract urothelial carcinomas. Hum. Pathol..

[34] Chaudhary D., Rath A., Mandal S., Khurana N., Agarwal P.N. (2022). Primary leiomyosarcoma kidney—A rare entity with a diagnostic challenge. J. Cancer Res. Ther..

[35] Choi J.H., Ro J.Y. (2020). Retroperitoneal Sarcomas: An Update on the Diagnostic Pathology Approach. Diagnostics.

[36] Kim T., Zargar-Shoshtari K., Dhillon J., Lin H.-Y., Yue B., Fishman M., Sverrisson E.F., Spiess P.E., Gupta S., Poch M.A. (2014). Using percentage of sarcomatoid differentiation as a prognostic factor in renal cell carcinoma. Clin. Genitourin. Cancer.

[37] Mian B.M., Bhadkamkar N., Slaton J.W., Pisters P.W.T., Daliani D., Swanson D.A., Pisters L.L. (2002). Prognostic factors and survival of patients with sarcomatoid renal cell carcinoma. J. Urol..

[38] Moch H., Amin M.B., Berney D.M., Compérat E.M., Gill A.J., Hartmann A., Menon S., Raspollini M.R., Rubin M.A., Srigley J.R. (2022). The 2022 World Health Organization Classification of Tumours of the Urinary System and Male Genital Organs-Part A: Renal, Penile, and Testicular Tumours. Eur. Urol..

[39] Delahunt B., Cheville J.C., Martignoni G., Humphrey P.A., Magi-Galluzzi C., McKenney J., Egevad L., Algaba F., Moch H., Grignon D.J. (2013). The International Society of Urological Pathology grading system for renal cell carcinoma and other prognostic parameters. Am. J. Surg. Pathol..

[40] International Collaboration on Cancer Reporting Datasets for Reporting Renal Epithelial Neoplasms/Carcinoma of Renal Tubular Origin. ICCR Structured Pathology Reporting Resources. https://www.iccr-cancer.org.

[41] Warren A.Y., Harrison D. (2018). WHO/ISUP classification, grading and pathological staging of renal cell carcinoma: Standards and controversies. World J. Urol..

[42] Samaratunga H., Gianduzzo T., Delahunt B. (2014). The ISUP system of staging, grading and classification of renal cell neoplasia. J. Kidney Cancer VHL.

[43] Chang A., Amin A., Gabrielson E., Epstein J.I. (2013). Use of PAX8 and GATA3 in diagnosing sarcomatoid renal cell carcinoma and sarcomatoid urothelial carcinoma. Hum. Pathol..

[44] Almuradova E., Basoglu T., Nayir E., Bayram E., Paydas S., Gokmen I., Karakaya S., Oksuzoglu B., Erdem D., Sakin A. (2023). Real-life experience of patients with sarcomatoid renal cell carcinoma: A multicenter retrospective study. Neoplasma.

[45] Schieda N., Thornhill R.E., Al-Subhi M., McInnes M.D.F., Shabana W.M., van der Pol C.B., Flood T.A. (2015). Diagnosis of Sarcomatoid Renal Cell Carcinoma with CT: Evaluation by Qualitative Imaging Features and Texture Analysis. Am. J. Roentgenol..

[46] Zhu H., Zhao S., Zuo C., Ren F. (2020). FDG PET/CT and CT Findings of Renal Cell Carcinoma with Sarcomatoid Differentiation. AJR Am. J. Roentgenol..

[47] Trudeau V., Larcher A., Sun M., Boehm K., Dell’oGlio P., Sosa J., Tian Z., Fossati N., Briganti A., Shariat S.F. (2016). Comparison of oncologic outcomes between sarcomatoid and clear cell renal cell carcinoma. World J. Urol..

[48] Shuch B., Said J., LaRochelle J.C., Zhou Y., Li G., Klatte T., Pouliot F., Kabbinavar F.F., Belldegrun A.S., Pantuck A.J. (2010). Histologic evaluation of metastases in renal cell carcinoma with sarcomatoid transformation and its implications for systemic therapy. Cancer.

[49] Nguyen D.P., Vilaseca A., Vertosick E.A., Corradi R.B., Touijer K.A., Benfante N.E., Sjoberg D.D., Russo P. (2015). Histologic subtype impacts cancer-specific survival in patients with sarcomatoid-variant renal cell carcinoma treated surgically. World J. Urol..

[50] Merrill M.M., Wood C.G., Tannir N.M., Slack R.S., Babaian K.N., Jonasch E., Pagliaro L.C., Compton Z., Tamboli P., Sircar K. (2015). Clinically non-metastatic renal cell carcinoma with sarcomatoid dedifferentiation: Natural history and outcomes after surgical resection with curative intent. Urol. Oncol.-Semin. Orig. Investig..

[51] Zhang B.Y., Thompson R.H., Lohse C.M., Leibovich B.C., Boorjian S.A., Cheville J.C., Costello B.A. (2014). A novel prognostic model for patients with sarcomatoid renal cell carcinoma. BJU Int..

[52] Mouallem N.E., Smith S.C., Paul A.K. (2018). Sarcomatoid renal cell carcinoma: Biology and treatment advances. Urol. Oncol.—Semin. Orig. Investig..

[53] Malouf G.G., Flippot R., Dong Y., Dinatale R.G., Chen Y.-B., Su X., Compérat E., Rouprêt M., Mano R., Blum K.A. (2020). Molecular scharacterisation of sarcomatoid clear cell renal cell carcinoma unveils new candidate oncogenic drivers. Sci. Rep..

[54] Bakouny Z., Braun D.A., Shukla S.A., Pan W., Gao X., Hou Y., Flaifel A., Tang S., Bosma-Moody A., He M.X. (2021). Integrative molecular scharacterisation of sarcomatoid and rhabdoid renal cell carcinoma. Nat. Commun..

[55] Bi M., Zhao S., Said J.W., Merino M.J., Adeniran A.J., Xie Z., Nawaf C.B., Choi J., Belldegrun A.S., Pantuck A.J. (2016). Genomic scharacterisation of sarcomatoid transformation in clear cell renal cell carcinoma. Proc. Natl. Acad. Sci. USA.

[56] Motzer R.J., Banchereau R., Hamidi H., Powles T., McDermott D., Atkins M.B., Escudier B., Liu L.F., Leng N., Abbas A.R. (2020). Molecular Subsets in Renal Cancer Determine Outcome to Checkpoint and Angiogenesis Blockade. Cancer Cell.

[57] Kim J., Ham W.S., Park J.S., Jang W.S. (2024). Incidence and Pattern of Recurrence after Surgical Resection in Organ-Confined Renal Cell Carcinoma. Yonsei Med. J..

[58] Wang L.L., Puri D., Saitta C., Liu F., Afari J.A., Meagher M.F., Hakimi K., Nguyen M.V., Shah A., Ghassemzadeh S. (2025). Trends and Outcomes in Sarcomatoid Renal Cell Carcinoma: Analysis of the National Cancer Data Base. Eur. Urol. Open Sci..

[59] Aeppli S., Engeler D.S., Fischer S., Omlin A., Pratsinis M., Hermann C., Rothermundt C. (2022). Incidence and outcome of patients with renal cell carcinoma treated with partial or radical nephrectomy in the Cantons St Gallen and Appenzell 2009–2018. Swiss Med. Wkly..

[60] Ji B., Li D., Fu S., Zhang Z., Yang T., Wu Y., Zuo Y., Xu Z., Yu N. (2020). Propensity-score matched comparison of partial versus radical nephrectomy for T1N0M0 sarcomatoid renal cell carcinoma. Transl. Androl. Urol..

[61] Vasudev N.S., Hutchinson M., Trainor S., Ferguson R., Bhattarai S., Adeyoju A., Cartledge J., Kimuli M., Datta S., Hanbury D. (2020). UK Multicenter Prospective Evaluation of the Leibovich Score in Localized Renal Cell Carcinoma: Performance has Altered Over Time. Urology.

[62] Mirza K.M., Taxy J.B., Antic T. (2016). Radical Nephrectomy for Renal Cell Carcinoma: Its Contemporary Role Related to Histologic Type, Tumor Size, and Nodal Status: A Retrospective Study. Am. J. Clin. Pathol..

[63] Silagy A.W., Mano R., Blum K.A., DiNatale R.G., Marcon J., Tickoo S.K., Reznik E., Coleman J.A., Russo P., Hakimi A.A. (2020). The Role of Cytoreductive Nephrectomy for Sarcomatoid Renal Cell Carcinoma: A 29-Year Institutional Experience. Urology.

[64] Feuerstein M.A., Kent M., Bernstein M., Russo P. (2014). Lymph node dissection during cytoreductive nephrectomy: A retrospective analysis. Int. J. Urol..

[65] Patel H.D., Karam J.A., Allaf M.E. (2018). Surgical Management of Advanced Kidney Cancer: The Role of Cytoreductive Nephrectomy and Lymphadenectomy. J. Clin. Oncol..

[66] Haas N.B., Lin X., Manola J., Pins M., Liu G., McDermott D., Nanus D., Heath E., Wilding G., Dutcher J. (2011). A phase II trial of doxorubicin and gemcitabine in renal cell carcinoma with sarcomatoid features: ECOG 8802. Med. Oncol..

[67] Michaelson M.D., McKay R.R., Werner L., Atkins M.B., Van Allen E.M., Olivier K.M., Song J., Signoretti S., McDermott D.F., Choueiri T.K. (2015). Phase 2 trial of sunitinib and gemcitabine in patients with sarcomatoid and/or poor-risk metastatic renal cell carcinoma. Cancer.

[68] Dutcher J.P., Nanus D. (2011). Long-term survival of patients with sarcomatoid renal cell cancer treated with chemotherapy. Med. Oncol..

[69] Escudier B., Droz J.P., Rolland F., Terrier-Lacombe M.J., Gravis G., Beuzeboc P., Chauvet B., Chevreau C., Eymard J.C., Lesimple T. (2002). Doxorubicin and ifosfamide in patients with metastatic sarcomatoid renal cell carcinoma: A phase II study of the Genitourinary Group of the French Federation of Cancer Centers. J. Urol..

[70] Choueiri T.K., Tomczak P., Park S.H., Venugopal B., Ferguson T., Chang Y.H., Hajek J., Symeonides S.N., Lee J.L., Sarwar N. (2021). Adjuvant Pembrolizumab after Nephrectomy in Renal-Cell Carcinoma. N. Engl. J. Med..

[71] Dibajnia P., Cardenas L.M., Lalani A.-K.A. (2023). The emerging landscape of neo/adjuvant immunotherapy in renal cell carcinoma. Hum. Vaccines Immunother..

[72] Pal S.K., Uzzo R., Karam J.A., Master V.A., Donskov F., Suarez C., Albiges L., Rini B., Tomita Y., Kann A.G. (2022). Adjuvant atezolizumab versus placebo for patients with renal cell carcinoma at increased risk of recurrence following resection (IMmotion010): A multicentre, randomised, double-blind, phase 3 trial. Lancet.

[73] Riveros C., Huang E., Ranganathan S., Klaassen Z., Rini B., Wallis C.J., Satkunasivam R. (2023). Adjuvant immunotherapy in renal cell carcinoma: A systematic review and meta-analysis. BJU Int..

[74] Choueiri T.K., Tomczak P., Park S.H., Venugopal B., Ferguson T., Symeonides S.N., Hajek J., Chang Y.H., Lee J.L., Sarwar N. (2024). Overall Survival with Adjuvant Pembrolizumab in Renal-Cell Carcinoma. N. Engl. J. Med..

[75] Eminaga O., Akbarov I., Wille S., Engelmann U. (2015). Does postoperative radiation therapy impact survival in non-metastatic sarcomatoid renal cell carcinoma? A SEER-based study. Int. Urol. Nephrol..

[76] Francolini G., Detti B., Ingrosso G., Desideri I., Becherini C., Carta G., Pezzulla D., Caramia G., Dominici L., Maragna V. (2018). Stereotactic body radiation therapy (SBRT) on renal cell carcinoma, an overview of technical aspects, biological rationale and current literature. Crit. Rev. Oncol. Hematol..

[77] A Stinauer M., Kavanagh B.D., E Schefter T., Gonzalez R., Flaig T., Lewis K., Robinson W., Chidel M., Glode M., Raben D. (2011). Stereotactic body radiation therapy for melanoma and renal cell carcinoma: Impact of single fraction equivalent dose on local control. Radiat. Oncol..

[78] Shuch B., Said J., La Rochelle J.C., Zhou Y., Li G., Klatte T., Kabbinaavar F.F., Pantuck A.J., Belldegrun A.S. (2009). Cytoreductive Nephrectomy for Kidney Cancer with Sarcomatoid Histology—Is Up-Front Resection Indicated and, if Not, is it Avoidable? *J*. Urol..

[79] Thomas A.Z., Adibi M., Slack R.S., Borregales L.D., Merrill M.M., Tamboli P., Sircar K., Jonasch E., Tannir N.M., Matin S.F. (2016). The Role of Metastasectomy in Patients with Renal Cell Carcinoma with Sarcomatoid Dedifferentiation: A Matched Controlled Analysis. J. Urol..

[80] Ouzaid I., Capitanio U., Staehler M., Wood C.G., Leibovich B.C., Ljungberg B., Van Poppel H., Bensalah K. (2019). Surgical Metastasectomy in Renal Cell Carcinoma: A Systematic Review. Eur. Urol. Oncol..

[81] Smith B.W., Joseph J.R., Saadeh Y.S., La Marca F., Szerlip N.J., Schermerhorn T.C., Spratt D.E., Younge K.C., Park P. (2018). Radiosurgery for Treatment of Renal Cell Metastases to Spine: A Systematic Review of the Literature. World Neurosurg..

[82] Lee-Ying R., Lester R., Heng D.Y. (2014). Current management and future perspectives of metastatic renal cell carcinoma. Int. J. Urol..

[83] Ueno T., Yamashita M., Sawada S., Sugimoto R., Nishijima N., Sugawara Y., Ninomiya I. (2016). Pulmonary metastasectomy from renal cell carcinoma including 3 cases with sarcomatoid component. Gen. Thorac. Cardiovasc. Surg..

[84] Ciccarese C., Büttner T., Cerbone L., Zampiva I., Monteiro F.S.M., Basso U., Pichler M., Vitale M.G., Fiala O., Roviello G. (2024). Clinical features and response to immune combinations in patients with renal cell carcinoma and sarcomatoid dedifferentiation (ARON-1 study). Int. J. Cancer.

[85] Zhi H., Feng M., Liu S., Na T., Zhang N., BiLiGe W. (2020). Prognostic Significance of Sarcomatoid Differentiation in Patients with Metastatic Renal Cell Carcinoma: A Systematic Review and Meta-Analysis. Front. Oncol..

[86] Iacovelli R., Ciccarese C., Bria E., Bracarda S., Porta C., Procopio G., Tortora G. (2020). Patients with sarcomatoid renal cell carcinoma—Re-defining the first-line of treatment: A meta-analysis of randomised clinical trials with immune checkpoint inhibitors. Eur. J. Cancer.

[87] Beecroft N., Gauntner T.D., Upadhyay R., Wang S.-J., Yang Y., A Singer E., Dason S. (2024). Active Surveillance in Metastatic Renal Cell Carcinoma. J. Kidney Cancer VHL.

[88] Fitzgerald K.N., Duzgol C., Knezevic A., Shapnik N., Kotecha R.R., Aggen D.H., Carlo M.I., Shah N.J., Voss M.H., Feldman D.R. (2023). Impact of sarcomatoid features on treatment outcomes in metastatic clear cell renal cell carcinoma treated with first-line immunotherapy combinations. J. Clin. Oncol..

[89] Kyriakopoulos C.E., Chittoria N., Choueiri T.K., Kroeger N., Lee J.-L., Srinivas S., Knox J.J., Bjarnason G.A., Ernst S.D., Wood L.A. (2014). Outcome of patients with metastatic sarcomatoid renal cell carcinoma: Results from the International Metastatic Renal Cell Carcinoma Database Consortium. Clin. Genitourin. Cancer.

[90] Rini B.I., Signoretti S., Choueiri T.K., McDermott D.F., Motzer R.J., George S., Powles T., Donskov F., Tykodi S.S., Pal S.K. (2022). Long-term outcomes with nivolumab plus ipilimumab versus sunitinib in first-line treatment of patients with advanced sarcomatoid renal cell carcinoma. J. Immunother. Cancer.

[91] McDermott D.F., Lee J.-L., Ziobro M., Suarez C., Langiewicz P., Matveev V.B., Wiechno P., Gafanov R.A., Tomczak P., Pouliot F. (2021). Open-Label, Single-Arm, Phase II Study of Pembrolizumab Monotherapy as First-Line Therapy in Patients with Advanced Non-Clear Cell Renal Cell Carcinoma. J. Clin. Oncol..

[92] Powles T., Plimack E.R., Soulières D., Waddell T., Stus V., Gafanov R., Nosov D., Pouliot F., Melichar B., Vynnychenko I. (2020). Pembrolizumab plus axitinib versus sunitinib monotherapy as first-line treatment of advanced renal cell carcinoma (KEYNOTE-426): Extended follow-up from a randomised, open-label, phase 3 trial. Lancet Oncol..

[93] McDermott D.F., Lee J.-L., Bjarnason G.A., Larkin J.M.G., Gafanov R.A., Kochenderfer M.D., Jensen N.V., Donskov F., Malik J., Poprach A. (2021). Open-Label, Single-Arm Phase II Study of Pembrolizumab Monotherapy as First-Line Therapy in Patients with Advanced Clear Cell Renal Cell Carcinoma. J. Clin. Oncol..

[94] Motzer R., Alekseev B., Rha S.-Y., Porta C., Eto M., Powles T., Grünwald V., Hutson T.E., Kopyltsov E., Méndez-Vidal M.J. (2021). Lenvatinib plus Pembrolizumab or Everolimus for Advanced Renal Cell Carcinoma. N. Engl. J. Med..

[95] Choueiri T.K., Larkin J.M.G., Pal S.K., Motzer R.J., Rini B.I., Venugopal B., Alekseev B., Miyake H., Gravis G., Bilen M. (2021). Efficacy and correlative analyses of avelumab plus axitinib versus sunitinib in sarcomatoid renal cell carcinoma: Post hoc analysis of a randomised clinical trial. ESMO Open.

[96] Choueiri T.K., Powles T., Burotto M., Escudier B., Bourlon M.T., Zurawski B., Oyervides Juárez V.M., Hsieh J.J., Basso U., Shah A.Y. (2021). Nivolumab plus Cabozantinib versus Sunitinib for Advanced Renal-Cell Carcinoma. N. Engl. J. Med..

[97] Grünwald V., Powles T., Eto M., Kopyltsov E., Rha S.Y., Porta C., Motzer R., Hutson T.E., Méndez-Vidal M.J., Hong S.H. (2023). Phase 3 CLEAR study in patients with advanced renal cell carcinoma: Outcomes in subgroups for the lenvatinib-plus-pembrolizumab and sunitinib arms. Front. Oncol..

[98] Motzer R.J., Penkov K., Haanen J., Rini B.I., Albiges L., Campbell M.T., Venugopal B., Kollmannsberger C., Negrier S., Uemura M. (2019). Avelumab plus axitinib versus sunitinib for advanced renal-cell carcinoma. N. Engl. J. Med..

[99] Rini B.I., Motzer R.J., Powles T., McDermott D.F., Escudier B., Donskov F., Hawkins R., Bracarda S., Bedke J., De Giorgi U. (2021). Atezolizumab plus Bevacizumab Versus Sunitinib for Patients with Untreated Metastatic Renal Cell Carcinoma and Sarcomatoid Features: Prespecified Subgroup Analysis of IMmotion151. Eur. Urol..

[100] Motzer R.J., Powles T., Atkins M.B., Escudier B., McDermott D.F., Alekseev B.Y., Lee J.L., Suarez C., Stroyakovskiy D., De Giorgi U. (2022). Final Overall Survival and Molecular Analysis in Metastatic RCC: IMmotion151. JAMA Oncol..

[101] Bergmann L., Albiges L., Ahrens M., Gross-Goupil M., Boleti E., Gravis G., Fléchon A., Grimm M.O., Bedke J., Barthélémy P. (2025). Prospective randomized phase-II trial of ipilimumab/nivolumab versus standard of care in non-clear cell renal cell cancer—Results of the SUNNIFORECAST trial. Ann. Oncol..

[102] Powles T., Albiges L., Bex A., Comperat E., Grünwald V., Kanesvaran R., Kitamura H., McKay R., Porta C., Procopio G. (2024). Electronic address: Clinicalguidelines@esmo.org. Renal cell carcinoma: ESMO Clinical Practice Guideline for diagnosis, treatment and follow-up. Ann. Oncol..

[103] Bex A., Ghanem Y.A., Albiges L., Bonn S., Campi R., Capitanio U., Dabestani S., Hora M., Klatte T., Kuusk T. (2025). European Association of Urology Guidelines on Renal Cell Carcinoma: The 2025 Update. Eur. Urol..

[104] Choueiri T.K., Albigès L., McDermott D.F., Hammers H.J., Escudier B., Burotto M., Plimack E.R., Porta C., George S., Powles T. (2026). Nivolumab plus ipilimumab versus sunitinib for first-line treatment of advanced renal cell carcinoma: Final analysis of efficacy and safety from the phase III CheckMate 214 trial. Ann. Oncol..

[105] Tannir N.M., Signoretti S., Choueiri T.K., McDermott D.F., Motzer R.J., Flaifel A., Pignon J.-C., Ficial M., Frontera O.A., George S. (2021). Efficacy and safety of nivolumab plus ipilimumab versus sunitinib in first-line treatment of patients with advanced sarcomatoid RCC. Clin. Cancer Res..

[106] Rini B.I., Plimack E.R., Stus V., Gafanov R., Hawkins R., Nosov D., Pouliot F., Alekseev B., Soulières D., Melichar B. (2019). Pembrolizumab plus axitinib versus sunitinib for advanced renal-cell carcinoma. N. Engl. J. Med..

[107] Motzer R.J., Escudier B., Burotto M., Powles T., Apolo A.B., Bourlon M.T., Shah A.Y., Porta C., Suárez C., Barrios C.H. (2025). Nivolumab plus cabozantinib (N+C) vs sunitinib (S) for previously untreated advanced renal cell carcinoma (aRCC): Final follow-up results from the CheckMate 9ER trial. J. Clin. Oncol..

[108] Choueiri T.K., Motzer R.J., Rini B.I., Haanen J., Campbell M.T., Venugopal B., Kollmannsberger C., Gravis-Mescam G., Uemura M., Lee J.L. (2020). Updated efficacy results from the JAVELIN Renal 101 trial: First-line avelumab plus axitinib versus sunitinib in patients with advanced renal cell carcinoma. Ann. Oncol..

[109] Hahn A.W., Surasi D.S., Viscuse P.V., Bathala T.K., Wiele A.J., Campbell M.T., Zurita A.J., Shah A.Y., Jonasch E., Gao J. (2024). Treatment Outcomes in Patients with Metastatic Renal Cell Carcinoma with Sarcomatoid and/or Rhabdoid Dedifferentiation After Progression on Immune Checkpoint Therapy. Oncologist.

